# Subcutaneous dirofilariasis due to *Dirofilaria immitis* in a dog in Brazil: first report

**DOI:** 10.1590/S1984-29612023032

**Published:** 2023-06-05

**Authors:** Welitânia Inácia da Silva, Alexander Rodrigo Dantas Gomes, Maria Carolina de Francisco, Janete Madalena da Silva, Hodias Sousa de Oliveira, Thais Ferreira Feitosa, Vinícius Longo Ribeiro Vilela

**Affiliations:** 1 Programa de Pós-graduação em Ciência e Saúde Animal, Universidade Federal de Campina Grande – UFCG, Patos, PB, Brasil; 2 PetVet Dr. Alexander Rodrigo, Natal, RN, Brasil; 3 Serviços em Patologia Veterinária, Belo Horizonte, MG, Brasil; 4 Programa de Pós-graduação em Ciência Animal, Universidade Federal da Paraíba – UFPB, Areia, PB, Brasil; 5 Departamento de Medicina Veterinária, Instituto Federal da Paraíba – IFPB, Sousa, PB, Brasil

**Keywords:** Dogs, microfilariae, ectopic parasitism, zoonosis, Cães, microfilárias, parasitismo ectópico, zoonose

## Abstract

The aim of this study was to report on the presence of microfilariae of *Dirofilaria immitis* causing nodular pyogranulomatous dermatitis in a dog in the state of Rio Grande do Norte, northeastern Brazil. A 4-year-old male dachshund dog with lesions in the nostrils and left dorsolateral regions was treated. Tests were requested to aid in making the diagnosis, such as skin cytology, Knott's test, thick smear and histopathology of the lesions. From these, presence of a diffuse pyogranulomatous process was observed and, amidst the cellular material, microfilariae of *Dirofilaria* spp. A conventional polymerase chain reaction test on tissue samples from the lesions revealed the presence of the species *D. immitis*. Treatment based on ivermectin (3mg) was administered at a single oral dose of 0.6 mg/kg. In the first seven days there was regression of the lesions, but after 30 days there was recurrence. A new treatment was administered, consisting of 10% imidacloprid + 2.5% moxidectin (4-10 mg/kg), with one application per month for 6 months, and doxycycline (100 mg), 10 mg/kg, 1 tablet, 2 times a day, for 30 days. In conclusion, *D. immitis* microfilariae caused pyogranulomatous lesions in the subcutaneous tissue of a dog. This had not previously been described in Brazil.

## Resumo

O objetivo deste trabalho foi relatar a presença de microfilárias de *Dirofilaria immitis* causando dermatite nodular piogranulomatosa em cão no estado do Rio Grande do Norte, região Nordeste do Brasil. Um cão de 4 anos, da raça Dachshund, foi atendido com lesões nas regiões das narinas e dorsolateral esquerda. Foram realizados exames de citologia cutânea, teste de Knott, gota espessa e histopatologia das lesões, sendo observada a presença de um processo piogranulomatoso difuso e microfilárias de *Dirofilaria* spp. Na reação em cadeia da polimerase convencional constatou-se a espécie *D. immitis* nas amostras de tecido das lesões. Foi efetuado um tratamento à base de Ivermectina (3mg) 0,6 mg/kg – 1 aplicação, via oral. Nos sete primeiros dias, houve regressão das lesões, porém, depois de 30 dias, houve recidiva. Foi efetuado um novo tratamento com imidacloprida 10% + moxidectina 2,5% (4-10 mg/kg), 1 aplicação por mês durante 6 meses, e Doxiciclina (100 mg), 10 mg/kg, 1 comprimido, duas vezes ao dia, por 30 dias. Em conclusão, microfilárias de *D. immitis* causaram lesões piogranulomatosas em tecido subcutâneo de um cão, relato anteriormente não descrito no Brasil.

Dirofilariasis is a disease with worldwide distribution caused by nematodes of the genus *Dirofilaria*. The species most often reported are *Dirofilaria immitis* and *Dirofilaria repens*, which both mainly infect dogs, although occurrences in cats, humans and wild carnivores have also been reported. The disease is transmitted through blood meals taken by hematophagous mosquitoes of the genera *Aedes*, *Anopheles* and *Culex* (Rodrigues et al., 2019; Dantas-Torres & Otranto, 2020).

In Brazil, the most prevalent and most studied species in dogs is *D. immitis*. In its adult form, this species affects the right ventricle and pulmonary artery and causes cardiopulmonary dirofilariasis (Labarthe et al., 2014). It is of importance with regard to One Health, given that it can affect humans through causing the formation of nodules in the lung parenchyma (Palicelli et al., 2022).

Subcutaneous dirofilariasis is more associated with the species *D. repens*. This disease remains poorly described but its distribution is mainly in Europe, Asia, Africa and, more recently, in the southern United States (Hays et al., 2020). Reports of subcutaneous lesions in dogs caused by *D. immitis* are rare. These consist of presence of multifocal ulcerative nodules or, in some cases, accidental findings in subcutaneous nodules, pelvic limbs or ocular tissue (Scott, 1979; Oliveira et al., 2021; Goh et al., 2023).

The treatment consists of the use of macrocyclic lactones as chemo preventive compounds. Among these, ivermectin is the compound most used, in association with doxycycline, and this treatment has been shown to reduce the risk of thromboembolism (Grandi et al., 2010). Furthermore, use of an agent based on imidacloprid and moxidectin in a single monthly dose for six months was shown to be effective for treating heartworms (Genchi et al., 2019).

The objective of the present study was to provide the first report from Brazil on the presence of *D. immitis* microfilariae causing nodular pyogranulomatous dermatitis in a dog.

A 4-year-old uncastrated male dachshund dog weighing 8 kg was admitted to a veterinary clinic in the city of Natal, state of Rio Grande do Norte, northeastern Brazil, at latitude 5° 44’ 46” S, longitude 35° 14’ 18” W. In the anamnesis, the dog’s keeper reported that, three months earlier, the animal had begun to present lesions in the nostril region that then progressed to cause respiratory difficulty. In the clinical examination, the vital parameters were within the reference values for the species, as described by Feitosa (2014).

**Figure 1:**
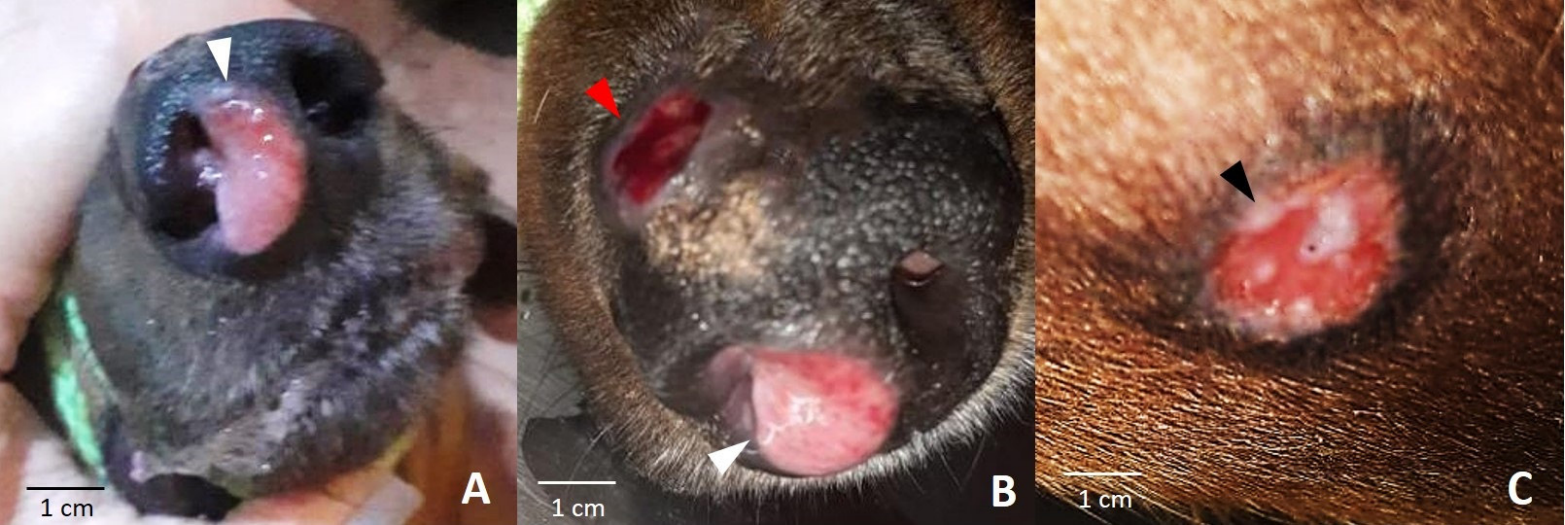
Uncastrated 4-year-old male dachshund dog showing skin lesions associated with *Dirofilaria immitis*. A-B. Ulcerated nodular lesion in the right nostril. Note a soft, shiny, pale pink nodule interspersed with discrete reddish areas, measuring approximately 2 x 1 cm (white arrowheads); B) Ulcerated circular lesion with a slightly depressed reddish center in the shape of a “nosepad”, measuring 2 x 2 cm (red arrowhead); C) Left dorsolateral region of the thorax, showing a circular area of alopecia, with a slightly depressed reddish ulcerated center, interspersed with white multifocal areas, measuring 2 x 2 cm (black arrowhead).

Through a specific physical examination, three skin lesions were observed: two around the nostrils and another on the left dorsolateral region (Figure 1). In the light of the animal’s clinical presentation, the initial suspicion was leishmaniasis, given that northeastern Brazil is an endemic region for this disease (Toepp et al., 2019). Thus, the serological tests of ELISA (Enzyme-Linked ImmunoSorbent Assay) and IFAT (ImmunoFluorescence Antibody Test). ELISA showed a non-reactive result; IFAT was also not reactive for the presence of anti-*Leishmania infantum* antibodies.

In addition to these tests, fragments of the lesions were collected in order to perform a conventional polymerase chain reaction (cPCR) test. For DNA extraction, the Purelink Genomic DNA mini-kit (Invitrogen®, California, United States) was used in accordance with the manufacturer's instructions. However, this cPCR test did not yield amplification of DNA from *L. infantum*.

Because of the nodular presentation of one of the lesions, a cutaneous neoplasm was suspected, and cytopathology and histopathology tests were requested. Through the cytological tests, intense cellularity of intact and degenerated neutrophils was observed along with, to a lesser extent, macrophages and red blood cells, in the presence of a pyogranulomatous inflammatory process.

**Figure 2:**
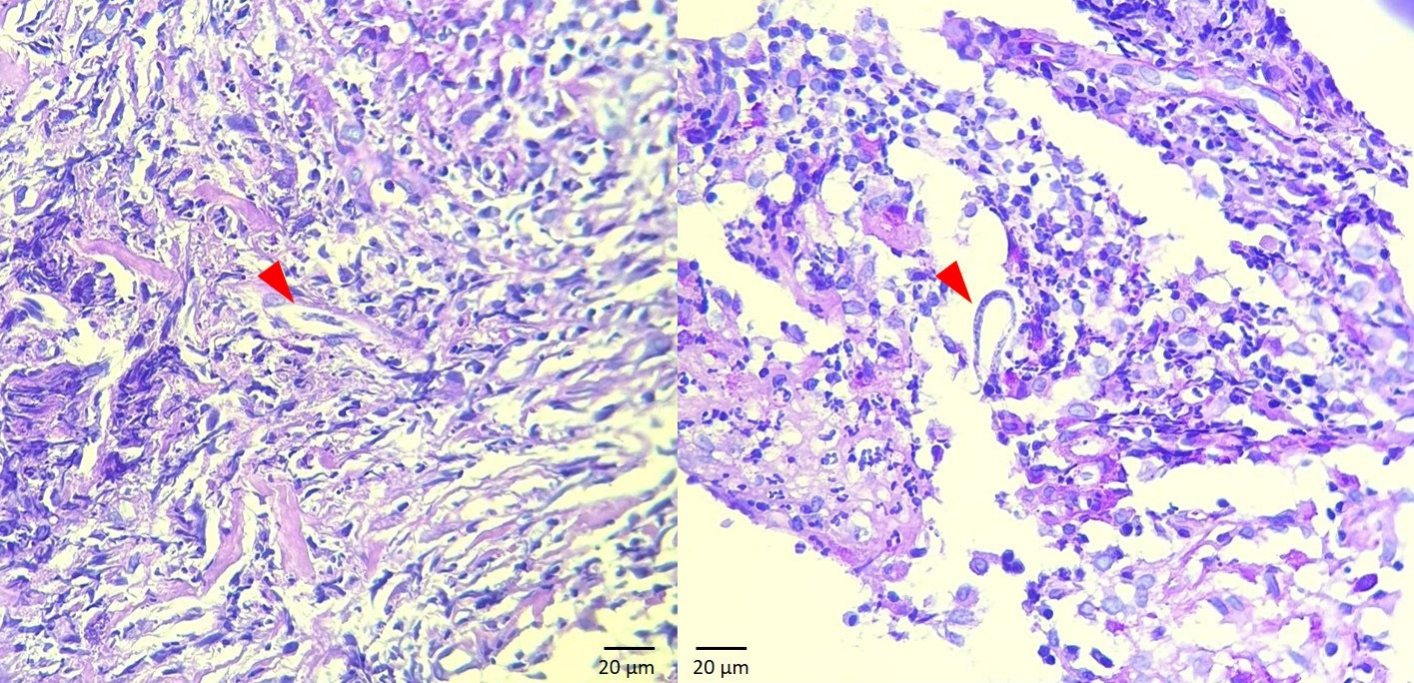
Histopathology of skin lesions associated with *D. immitis* infection in an uncastrated 4-year-old male dachshund dog. The superficial and deep dermis present pyogranulomatous inflammatory infiltrate, composed predominantly of macrophages, neutrophils and lymphocytes, with plasmocytes, mast cells and eosinophils in smaller quantities, mixed with areas of suppuration. Among the inflammatory cells, elongated structures morphologically compatible with filarial nematodes can be observed (red arrowheads). H&E. 400X magnification.

For the histopathological examination, from each lesion, a soft and whitish cylindrical skin fragment measuring on average 0.8 x 0.3 x 0.3 cm was collected using a 04 punch and immersed in 10% formaldehyde. Sections cut from the fragments showed that the epidermis was intact and irregular, with hyperkeratosis and foci of discrete pigment effusion. PAS (Periodic Acid-Schiff) staining showed that the samples were negative for visualization of fungi, and BAAR (Acid-Alcohol Resistant Bacilli) staining showed that they were negative for acid-alcohol resistant bacteria. However, rare small and elongated larval forms were observed amidst the inflammatory material, in the presence of nodular pyogranulomatous dermatitis of parasitic etiology (Figure 2).

These findings led to a new suspicion of dirofilariasis. Thus, whole-blood samples were collected to carry out additional thick blood tests. In these tests, circulating larvae of the parasite could be seen in the midst of cellular material. The modified Knott test (Bowman, 2010) was then used to perform morphometric analysis on microfilariae. The test result was read using a LAB-DM300 digital optical microscope, coupled to a computer equipped with photomicrograph software, using 40× and 100× lenses (400× and 1,000× magnification). Measurements were made using the Mv Image® software tools. It was observed that the microfilariae had a slightly tapered anterior end, conical head, straight tail, absence of a cephalic hook, average length of 205-283 μm and average width of 6-6.5 μm, respectively, according to Companion Vector-Borne Diseases (CVBD, 2006), indicating that they are L1 of *D. immitis* (Bowman, 2010).

To confirm the parasite species, tissue fragments from the three lesions had previously been sent to MBL for analysis. For DNA extraction, the Purelink Genomic DNA mini-kit (Invitrogen®, California, United States) was used in accordance with the manufacturer's instructions. The samples were then subjected to cPCR using the primers described by Oh et al. (2017), which were specific for *D. immitis* (forward: 5′ -ATT GGG TGC CCC TGA AAT GG -3′ and reverse: 5′ -CCC TCT ACA CTC AAA GGA GGA -3′). These amplified a 150-bp fragment on 2% agarose gel. A sample known to be positive for the DNA of *D. immitis* was used as a positive control, and ultrapure water (MilliQ®) was used as a negative control. There was amplification of the DNA of *D. immitis* in the analysed samples.

In view of the diagnosis of subcutaneous heartworm caused by *D. immitis*, the initial treatment instituted consisted of the following: ivermectin (Mectimax® 3mg), 0.6 mg/kg orally, with an application interval of 30 days (Bowman & Mannella, 2011); prednisone (Meticorten® 5 mg), 1mg/kg p.o. BID, for 5 days; and cephalexin (Keflex® 250 mg/5mL), 15 mg/kg p.o. BID, for 10 days (Bowman & Atkins, 2009).

The dog was brought back to the clinic 30 days after the beginning of the treatment, with the complaint that the lesions had evolved. Therefore, a new treatment was instituted, consisting of 10% imidacloprid + 2.5% moxidectin (Advocate®4-10 mg/kg), applied once a month, for six months, in association with doxycycline (Doxitec® 100 mg), 10 mg/kg, one tablet SID for 30 days (Jacobson & DiGangi, 2021). After the second treatment, there was total regression of the lesions, and the Knott and thick smear tests became negative for microfilariae.

Subcutaneous dirofilariasis is most often associated with the nematode *D. repens*, which is endemic in the Old World (Anvari et al., 2020). In the Americas, there have been reports of *D. immitis* causing subcutaneous lesions in dogs in New Jersey and South Carolina, USA (Scott, 1979; Oliveira et al., 2021). Northeastern Brazil is an endemic region for heartworm disease caused by *D. immitis*, and occurrences of cardiopulmonary disease in dogs through this cause are common (Labarthe et al., 2014). The dog in this report was living in a coastal area in northeastern Brazil, which has a hot and humid tropical climate (Reifur et al., 2004) that is favorable for occurrence of infections due to this parasite. However, this was the first report in this country of a dog that was affected by *D. immitis* and presented nodular pyogranulomatous subcutaneous lesions.

The pathogenesis of subcutaneous dirofilariasis is still not well understood, and it is not known whether the lesions are caused directly by the action of nematodes through capillary embolization by microfilariae, or as a consequence of a hypersensitivity reaction that evolves into a chronic inflammatory condition (Rocconi et al., 2012). Cases of ectopic migration of *D. immitis* in cutaneous tissues causing aggravating dermatological alterations are not common. In the case reported here, the lesions were in the nostrils and left dorsolateral region, and these evolved into nodular pyogranulomatous dermatitis. No explanation for the occurrence of these ectopic migrations has yet been reached, but they can be found in the brain, spinal cord, eyes, peritoneal cavity and skin tissue, and are more common in cats than in dogs (Oh et al., 2008). In Brazil, there is one report of a cat co-infected by *Dioctophyme renale* and *Dirofilaria* sp., found in a nodule in subcutaneous tissue (Vidal et al., 2021).

The clinical presentation of subcutaneous dirofilariasis caused by *D. repens* is associated with the presence of alopecia, pruritus, erythema, papular or nodular dermatitis and/or panniculitis (Paździor-Czapula et al., 2018) Thus, involvement of *D. repens* was suspected, given that the dog presented ulcerated nodular lesions in the region of the nostrils and left dorsolateral area, with areas of alopecia. However, no presence of pruritus was reported by the owner. These features demonstrate that the cutaneous and subcutaneous lesions were like those caused by *D. repens*. Scott (1979) observed that ulcerative cutaneous nodules found in three dogs were associated with the presence of *D. immitis*, which may further emphasize the similarity between these two species of *Dirofilaria* spp. Therefore, it is essential to carry out tests that distinguish between the two species, in seeking to make a definitive diagnosis.

Histologically, skin lesions associated with *Dirofilaria* spp. are characterized by a pyogranulomatous or granulomatous inflammatory process, with the presence of eosinophils, lymphocytes, plasma cells and some mast cells, in association with microfilariae or, occasionally, intralesional adult nematodes (Scott, 1979; Paździor-Czapula et al., 2018). In the case reported here, the histopathological examination on the skin and nasal plane lesions revealed pyogranulomatous dermatitis, with predominance of typical macrophages, neutrophils and lymphocytes. Plasma cells, mast cells and eosinophils were observed in smaller quantities, in association with the presence of intralesional microfilariae. The absence of intralesional adult nematodes in the tissue samples evaluated does not rule out the possibility that they were present in other areas of the lesions. In the present report, the definitive diagnosis was made through histopathology, together with cPCR on fragments from the skin lesions.

The initial treatment based on ivermectin every 30 days, as described by Bowman & Mannella (2011), for five days was not effective in eliminating the microfilariae, and the dog was brought back to the clinic with recurrence of the lesions after this first therapeutic protocol. Prescription of another, more prolonged treatment was required. According to Giannelli et al. (2013), use of ivermectin alone is not effective, in that it only kills filariids for a short period. Thus, a treatment that is more effective is required for complete elimination of microfilaremia and adult nematodes. Genchi et al. (2019) conducted a study in which they treated dogs with heartworm disease with a topical formulation containing 10% imidacloprid and 2.5% w/v moxidectin (Advocate®, Advantage Multi®, Bayer), monthly for nine months, in association with doxycycline (10 mg/kg BID), observing a microfilaremia reduction after 30 days and negative antigens for *D. immitis* after nine months. In the case reported here, medications based on imidacloprid 10% + moxidectin 2.5% (Advocate® 4-10 mg/kg) were prescribed, with one application per month for six months, along with doxycycline 10 mg/kg, one tablet SID, for 30 days (Doxitec®100 mg). This led to complete regression of the lesions, thus demonstrating the efficacy of the second treatment.

In conclusion, *D. immitis* microfilariae were found to cause pyogranulomatous lesions in the subcutaneous tissue of a dog. This was the first report of this occurrence in Brazil. *D. immitis* is an important parasite regarding One Health, given that it has zoonotic potential. This report of its occurrence in an ectopic site is of great value, considering that northeastern Brazil is an endemic area for heartworm disease. Thus, this report signals to veterinarians that they should include this parasitic agent as a differential diagnosis in cases of ulcerative skin lesions.
